# Appeal in Behalf of the Wells National Testimonial Fund

**Published:** 1872-09

**Authors:** 


### ARTICLE III.

*Appeal in Behalf of the Wells National Testimonial Fund.*

It is proposed to raise a fund for the benefit of the family of the late Horace Wells, the discoverer of the principle of Anæsthesia, or the prevention of pain in surgical and dental operations, by the inhalation of nitrous oxide gas and other agents.

Dr. Wells was a benefactor to the world.

He conferred upon it the physical boon, in the prevention of pain in severe operations in general surgery and obstetrics, as well as in minor operations in dentistry. Without its aid, many of them would be impossible, while with it they have not only been rendered possible, but, directly and indirectly, tens of thousands of lives, as well as an untold amount of mental and physical anguish, has been saved. To accomplish this, his own health, life and fortune were sacrificed in his zeal for the perfection of his great discovery, and his early death left those whom he loved and cherished unprovided with even the most moderate means of support.

This appeal is therefore made, in behalf of *his family*, to the members of the dental profession, as well as to private citizens, benefited directly or indirectly by his great discovery, to aid in making up a Testimonial Fund as an act of tardy justice to the memory of an American citizen who deserves to rank with Morse and Fulton among the world's benefactors; and whose claims to everlasting and grateful remembrance is enhanced by the fact that *he loved his fellow-men better than himself.*

We would, therefore, urge the dental profession to embrace this opportunity, and generously show their appreciation of Horace Wells and his discovery, and of the immense benefit it has been to them, by contributing to this fund.

Subscriptions may be sent direct to the Treasurer of the Fund, to whom all contributions will be paid over.

PHILADELPHIA:—Louis Jack, D. D. S.; Daniel Neal, D. D. S.; Geo. T. Barker, D. D. S.; J. H. McQuillen, D. D. S.; E. Wildman, D. D. S.; D. D. Smith, D. D. S.

BOSTON:—Geo. T. Moffat, M. D.; N. W. Hawes, M. D.; Thos. B. Hitchcock, M. D.; Thos. H. Chandler, M. D.; Jacob L. Williams, M. D.

NEW YORK:—William H. Allen; Frank Abbot, M. D.; J. S. Latimer, D. D. S.; Norman W. Kingsley, D. D. S.

BALTIMORE:—F. J. S. Gorgas, D. D. S.

TREASURER, Samuel S. White, Chestnut cor. Twelfth Sts., Philadelphia. 767 & 769 Broadway, New York. 13 & 16 Tremont Row, Boston. 14 & 16 Madison St., Chicago.

SECRETARY, Dental Profession, James Trueman, D. D. S., 1221 Spruce Street, Philadelphia.

---

